# Effect of Perioperative Tricyclic Antidepressants and First-Generation Antihistamines on Postoperative Delirium in Older Patients Undergoing Arthroplasty or Spine Surgery

**DOI:** 10.7759/cureus.105447

**Published:** 2026-03-18

**Authors:** Adrian Jones, Laura Sands, Li Zhang, Jacqueline Leung, Marc Buren

**Affiliations:** 1 Anesthesiology, University of California Los Angeles, Los Angeles, USA; 2 Department of Human Development and Center for Gerontology, Virginia Tech, Blacksburg, USA; 3 Epidemiology and Biostatistics, University of California San Francisco, San Francisco, USA; 4 Anesthesia and Perioperative Care, University of California San Francisco, San Francisco, USA

**Keywords:** anticholinergics, antihistamines, major orthopedic surgery, orthopedic surgery, postoperative delirium, tricyclic antidepressants

## Abstract

Background

Postoperative delirium (POD) is a prevalent condition in older surgical patients, leading to extended hospital stays and increased risk of cognitive and functional decline. We investigated whether in-hospital perioperative administration of tricyclic antidepressants (TCAs) or first-generation antihistamines, both of which have potent anticholinergic activity, was associated with postoperative delirium within the first three postoperative days in older surgical patients undergoing major arthroplasty or spine surgeries.

Methods

We undertook a secondary data analysis of patients aged 65 or older staying in the hospital for at least two days undergoing hip, knee, or spine surgeries from two prospective studies. Using propensity score matching applied separately for each medication class (TCAs and first-generation antihistamines) to respective control groups. Our primary outcome was the incidence of postoperative delirium within the first three postoperative days, as gauged by the Confusion Assessment Method.

Results

After propensity score matching, which was applied separately for each medication class, no difference in demographics, geriatric depression scores, cognitive status scores, or surgical details emerged between the medication recipients and non-recipients. The perioperative use of tricyclic antidepressants (OR: 1.82, 95% CI: 0.78-4.16) or first-generation antihistamines (OR: 0.79, 95% CI: 0.48-1.28) was not statistically significantly associated with postoperative delirium.

Conclusion

In this secondary analysis of older patients undergoing elective major arthroplasty or spine surgery, we did not detect a statistically significant association between perioperative tricyclic antidepressant or first-generation antihistamine use and postoperative delirium within the first three postoperative days. These findings should be interpreted cautiously, given the limited sample size for TCA exposure.

## Introduction

Postoperative delirium (POD) is a geriatric syndrome characterized by acute changes in attention, cognition, and/or awareness that may fluctuate in the days after surgery [1]. POD is associated with prolonged hospital stay, increased risk of functional and cognitive decline, and placement in facilities other than home after discharge [2-4]. It is hypothesized that POD is precipitated by an interaction between predisposing and precipitating factors. Predisposing factors include advanced age, baseline cognitive dysfunction, and multiple comorbidities, whereas precipitating factors are thought to include perioperative pain, sleep-wake disturbances, blood transfusions, and increased surgery duration [1,5].

Older patients are more prone to adverse effects of certain classes of medications [6]. Potentially inappropriate medications (PIM) are delineated by the American Geriatrics Society (AGS) in the Beers criteria, which lists medications that should be avoided or used with caution in older adults [7]. Current practice guidelines recommend avoiding these medications in older adults undergoing major surgery to prevent postoperative delirium [8]. While the Beers medications list is important, definitive data supporting the underlying recommendations for avoidance of use by older patients are limited.

An important category of medications on the Beers list includes drugs with anticholinergic activity. Anticholinergic medications can lead to a deficiency in central nervous system acetylcholine, a neurotransmitter that plays an important role in memory and consciousness [9,10]. Both first-generation H-1 antihistamines and tricyclic antidepressants (TCAs) are included on the Beers list and possess potent anticholinergic activity. These medications are frequently prescribed chronically and are often continued or administered in the perioperative period; however, data specifically evaluating their independent association with POD are limited. TCAs are commonly used for the treatment of major depressive disorder and can also be used off-label for migraine prophylaxis, obsessive-compulsive disorder, insomnia, anxiety, and chronic pain [11]. Antihistamines that bind to H-1 receptors are generally used to treat allergic rhinitis and allergies, but are commonly used in the postoperative period as a sleep aid and for the management of postoperative nausea and vomiting. The central H-1 effects in first-generation agents mediate the common side effect of sedation and can lead to altered sleep-wake cycles, while additional anticholinergic activity could lead to effects on cognition [12]. TCAs and first-generation antihistamines are strongly recommended to be avoided in older adults [8]. Prior research has largely focused on composite anticholinergic burden scores rather than specific medication classes, and findings have been mixed, with some studies reporting an association between higher cumulative anticholinergic exposure and POD, while others report no independent association after accounting for baseline cognitive impairment and comorbidity. These findings differ from our results and suggest that cumulative anticholinergic exposure across multiple medications may be more clinically relevant than exposure to an individual drug class in isolation.

In addition, burden scores may capture additive effects across multiple medications, whereas analyses of individual drug classes may miss clinically important cumulative exposure [13]. Emerging evidence also suggests that perioperative anticholinergic exposure may be associated with longer-term cognitive outcomes, including incident dementia, underscoring the broader clinical importance of understanding their perioperative effects [14]. 

To further investigate the association between the perioperative administration of anticholinergic medications and POD, we performed a secondary observational analysis of data derived from two prospective studies using propensity score matching applied separately for each medication class to examine the association between in-hospital perioperative administration of TCAs or first-generation antihistamines and the occurrence of POD in patients aged 65 and older undergoing major arthroplasty or spine surgeries. We hypothesized that perioperative exposure to these anticholinergic medication classes would be associated with an increased risk of POD.

## Materials and methods

Study design and population

The study was approved by the University of California, San Francisco (UCSF) Institutional Review Board (IRB). Written informed consent was obtained from all subjects. This study represents a secondary analysis of prospectively collected data from two studies conducted at the UCSF Medical Center between 2005 and 2014. Both parent studies enrolled patients aged ≥65 years undergoing major arthroplasty or spine surgery. The first study was a randomized controlled trial of 750 patients assigned to receive perioperative gabapentin or placebo (ClinicalTrials.gov identifier: NCT00221338) [15]. The parent randomized trial was included in this secondary analysis because the primary outcome (POD incidence) did not differ between the intervention and control groups. The second study was a prospective observational cohort study of 499 patients investigating the pathophysiology of POD [16,17]. Inclusion criteria for this second prospective study were patients aged ≥65 years old, scheduled for major noncardiac surgery at UCSF Medical Center, fluent in English, and had an expected hospital stay of at least 48 hours after surgery. To minimize variability due to surgery type, patients who underwent procedures other than major arthroplasty or spine surgeries were excluded. Although the two studies differed slightly in expected length-of-stay criteria (≥72 hours versus ≥48 hours), both enrolled patients aged ≥65 years undergoing major arthroplasty or spine surgery and used identical delirium assessment protocols, permitting harmonized secondary analysis.

Patients were eligible for inclusion in the current analysis if they received TCAs or first-generation antihistamines on the day of surgery (DOS), intraoperatively, or during the first three postoperative days. Medication exposure was defined as any documented administration during this perioperative window; information regarding dosage, frequency, and cumulative exposure was not available. Patients were excluded if they underwent brain or cardiac surgery or were unable to provide written informed consent. To minimize variability in surgical risk, only patients undergoing major arthroplasty or spine surgery were included. This manuscript is reported in accordance with the Strengthening the Reporting of Observational Studies in Epidemiology (STROBE) guidelines [18].

Preoperative assessment

Clinical Information

Patient's demographic information and medical history of coexisting diseases were extracted from the medical records. Physical status and anesthesia risk were measured by the American Society of Anesthesiologists (ASA) physical status [19]. Severity of preoperative coexistent conditions was determined using the Charlson comorbidity index [20].

Data for the Study

Preoperative assessment was conducted by trained research assistants in the preoperative anesthesia clinic or over the telephone, typically performed within one week of surgery. Preoperative cognitive status was measured in all patients using the Telephone Interview for Cognitive Status (TICS), an 11-item screening test that was adapted from the Mini-Mental State Examination for use in person or over the phone [21]. Scores range from zero to 41 points, and a TICS score of 31 or less was used to classify patients as having cognitive impairment [22]. The Geriatric Depression Scale (GDS) consists of 15 questions, and the total score is the sum of depressive symptoms. Scores of five or greater indicate moderate to severe depression [23]. TCAs that were present in our data set included nortriptyline, imipramine, doxepin, desipramine, clomipramine, and amitriptyline. First-generation antihistamines present in our data set included chlorpheniramine, diphenhydramine, hydroxyzine, meclizine, and promethazine. Perioperative is defined as the DOS, intraoperatively, or during the first three postoperative days.

Outcome definition

The primary outcome was the incidence of postoperative delirium (POD) observed in any of the first three postoperative days. Structured interviews were conducted in person by trained research assistants to determine the occurrence of POD using the Confusion Assessment Method (CAM), typically one week prior to surgery, and then once daily on the first three postoperative days, and were identical between both prospective trials. Assessments were conducted at approximately the same time each postoperative day. The CAM is a screening tool for detecting delirium adapted from the DSM-5 diagnostic criteria, with high sensitivity, specificity, and interrater reliability [24]. The CAM algorithm consists of four clinical criteria: 1) acute onset and fluctuating course of mental status, 2) inattention, 3) disorganized thinking, and 4) altered level of consciousness. For delirium to be recorded, both the first and second criteria must be present, plus either criterion three or four. All cases of delirium determined by the research assistants were validated by a second investigator (Jacqueline Leung or Laura Sands). We chose three days as the cutoff, as most of our patients were discharged in the early postoperative period. Patients with tracheal intubation who were unable to fully participate in CAM screening were excluded.

Statistical analysis

Because this was a secondary analysis of prospective data, we did not preregister our protocol, and a data analysis and statistical plan were written after the data were accessed. Two separate sets of bivariate analyses were performed to compare the characteristics of patients who received perioperative TCAs and those who received first-generation antihistamines, versus those who did not receive either TCAs or first-generation antihistamines. Mann-Whitney U tests were used to compare the continuous variables between two groups. Chi-squared tests were used to compare the categorical variables between the groups. All statistical analyses were conducted using R (R version 4.0.5; R Foundation for Statistical Computing, Vienna, Austria). Statistical significance was declared based on a p-value <0.05. Patients with missing delirium outcome data or missing covariates required for propensity score generation were excluded from matched analyses; no imputation was performed.

We used propensity scores to construct two matched control groups by matching each patient receiving the perioperative medication of interest, TCAs or first-generation antihistamines, to untreated patients with similar baseline characteristics. Propensity scores were generated separately for the TCA and antihistamine analyses to estimate the probability of receiving the medication of interest based on preoperative characteristics, including sex, age, GDS score, TICS score, ASA class, and surgical site. Patients receiving perioperative TCAs were matched 1:4 with controls, and patients receiving first-generation antihistamines were matched 1:1 with controls. Control patients did not receive the medication of interest, and patients serving as controls in the TCA analysis were not included in the antihistamine control group, and vice versa, to avoid exposure overlap.

Propensity score matching was performed using the MatchIt package in R with nearest-neighbor matching. Covariate balance after matching was assessed using standardized mean differences (SMD), with SMD <0.1 considered indicative of adequate balance. After matching, univariable logistic regression was used to evaluate the association between medication exposure and POD within the first three postoperative days. Variables demonstrating residual imbalance after matching (SMD >0.1) were included along with the medication exposure variable in multivariable logistic regression models to account for remaining confounding [25].

## Results

Patient population

**Figure 1:**
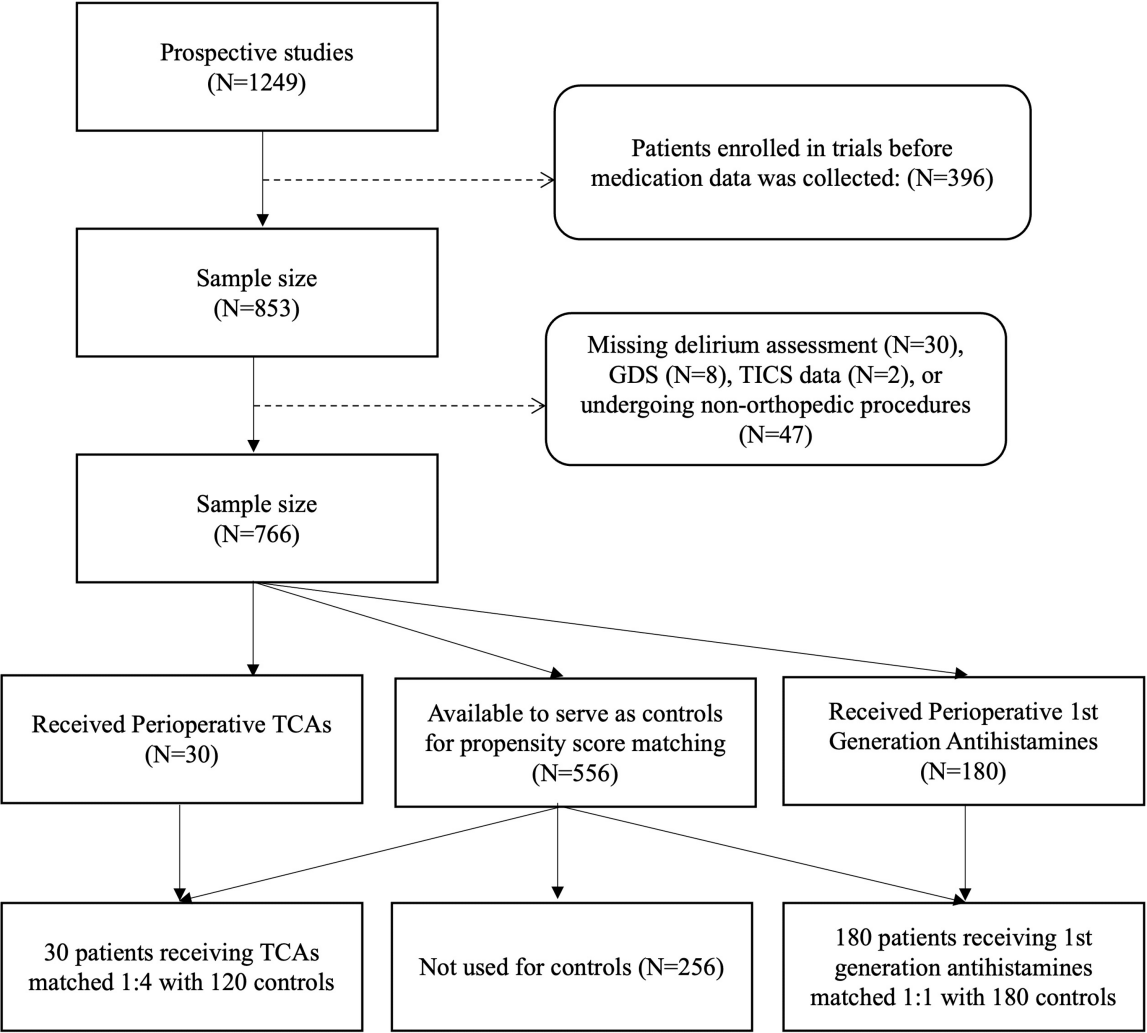
Flow chart for the inclusion of patients in the propensity score analyses GDS - Geriatric Depression Scale; TICS - Telephone Interview for Cognitive Status

Three hundred and ninety-six patients were enrolled in the two prospective studies before medication data were collected, and were excluded from this study. Thirty patients were excluded due to incomplete or missing delirium data. The reasons for missing delirium data were due to patients' refusal of assessment, sedation status, or patients who remained intubated postoperatively. Eight patients had incomplete GDS data, two patients had incomplete TICS score data, and 47 patients were excluded for undergoing non-orthopedic or spine surgeries. A total of 766 patients were available for propensity score matching. A flowchart for inclusion in the propensity score analytic sample is shown in Figure 1.

Patient characteristics of perioperative TCA usage

Clinical characteristics for the propensity-matched cohorts of patients who received or did not receive TCAs at any time during the in-hospital perioperative period are presented in Table 1. POD occurred in 13 of 30 patients (43%) receiving TCAs compared with 35 of 120 matched controls (29%). After propensity score matching, those who did and did not receive TCAs had no difference in their mean age, gender, GDS score, TICS score, ASA class, or surgical site. Thirty patients received TCAs at any point perioperatively with POD measurements and were matched 1:4 with 120 controls, for a total of 150 patients included in this analysis. Based on the propensity score matching data, we performed both univariable and multivariable analyses to assess if any variables were associated with the occurrence of POD within the first three days following surgery (Table 2). The perioperative use of TCAs was not significantly associated with POD, OR: 1.82 (95% CI: 0.78-4.16, p=0.16). The TCAs included in the study were nortriptyline (n=3), imipramine (n=2), doxepin (n=3), desipramine (n=2), clomipramine (n=1), and amitriptyline (n=19).

**Table 1:** Preoperative demographic data of TCA propensity-matched cohorts TCA - tricyclic antidepressant; SMD - standardized mean difference; GDS - Geriatric Depression Scale; TICS - Telephone Interview for Cognitive Status; ASA - American Society of Anesthesiologists

Variable	TCA use (n=30)	No TCA use (n=120)	SMD	Test statistic
Age, median (IQR)	70 (68-73)	70 (67-75)	0.04	Mann–Whitney U=1702.5
Gender				
Female	22 (73%)	93 (78%)	0.1	χ²(1)=0.29
Male	8 (27%)	27 (23%)		
GDS				
<5	15 (50%)	60 (50%)	<0.001	χ²(1)=0.00
≥5	15 (50%)	60 (50%)		
TICS				
>31	22 (73%)	94 (78%)	0.12	χ²(1)=0.34
≤31	8 (27%)	26 (22%)		
ASA class				
≤2	16 (53%)	67 (56%)	0.05	χ²(1)=0.08
≥3	14 (47%)	53 (44%)		
Surgical site				
Hip	4 (13%)	18 (15%)	0.05	χ²(2)=0.07
Knee	7 (23%)	28 (23%)		
Spine	19 (63%)	74 (62%)		

**Table 2:** Association of variables associated with the occurrence of POD within the first three days following surgery after propensity score matching for TCA use TCA - tricyclic antidepressant; POD - postoperative delirium; GDS - Geriatric Depression Scale; TICS - Telephone Interview for Cognitive Status; ASA - American Society of Anesthesiologists

Variable	No POD (n=102)	POD (n=48)	Univariable OR (95% CI; z, p)	Multivariable OR (95% CI; z, p)
Age, median (IQR)	70 (67-74)	71 (67-78)	1.05 (0.99-1.11; z=1.51, p=0.13)	-
Gender				
Female	77 (76%)	38 (79%)		
Male	25 (25%)	10 (21%)	0.81 (0.34-1.82; z=-0.50, p=0.62)	-
GDS				
<5	55 (54%)	20 (42%)		
≥5	47 (46%)	28 (58%)	1.64 (0.82-3.31; z=1.41, p=0.16)	-
TICS				
>31	83 (81%)	33 (69%)		
≤31	19 (19%)	15 (31%)	1.99 (0.90-4.37; z=1.70, p=0.09)	1.95 (0.87-4.32; z=1.64, p=0.10)
ASA class				
≤2	58 (57%)	25 (52%)		
≥3	44 (43%)	23 (48%)	1.21 (0.61-2.42; z=0.55, p=0.58)	-
Surgical site				
Hip	17 (17%)	5 (10%)		-
Knee	24 (24%)	11 (23%)	1.56 (0.47-5.70; z=0.71, p=0.48)	
Spine	61 (60%)	32 (67%)	1.78 (0.64-5.82; z=1.04, p=0.30)	
Perioperative TCA use				
No	85 (83%)	35 (73%)		
Yes	17 (17%)	13 (27%)	1.86 (0.81–4.22; z=1.48, p=0.14)	1.82 (0.78–4.16; z=1.41, p=0.16)

Patient characteristics of perioperative first-generation antihistamine usage

Clinical characteristics for the propensity-matched cohorts of patients who received or did not receive first-generation antihistamines at any time during the in-hospital perioperative period are presented in Table 3. POD occurred in 39 of 180 patients (22%) receiving first-generation antihistamines compared with 48 of 180 matched controls (27%). After propensity score matching, those who did and did not receive first-generation antihistamines had no difference in their mean age, gender, GDS score, TICS score, ASA class, or surgical site. One hundred and eighty patients received first-generation antihistamines at any point perioperatively with POD information and were matched 1:1 with controls, with 360 total patients in this analysis. Based on the propensity score matching data, we performed both univariable and multivariable analyses to assess if any variables were associated with the occurrence of POD within the first three days following surgery (Table 4). Higher GDS, OR: 2.36 (95% CI: 1.39-3.99, p=0.001), lower TICS, OR: 2.11 (95% CI: 1.10-3.99, p=0.02), and higher ASA status, OR: 1.82 (95% CI: 1.12-2.97, p=0.02), were associated with a higher chance of developing POD. The perioperative use of first-generation antihistamines was not significantly associated with POD, OR: 0.79 (95% CI: 0.48-1.28, p=0.33). The first-generation antihistamines included in this study were chlorpheniramine (n=3), diphenhydramine (n=133), hydroxyzine (n=43), meclizine (n=6), and promethazine (n=18). Twenty-three patients received more than one first-generation antihistamine.

**Table 3:** Preoperative demographic data of first-generation antihistamine propensity-matched cohorts SMD - standardized mean difference; GDS - Geriatric Depression Scale; TICS - Telephone Interview for Cognitive Status; ASA - American Society of Anesthesiologists

Variable	Antihistamine use (n=180)	No antihistamine use (n=180)	SMD	Test statistic
Age, median (IQR)	70 (67-74)	70 (67-74)	0.06	Mann–Whitney U=15975.5
Gender				
Female	110 (61%)	119 (66%)	0.1	χ²(1)=1.01
Male	70 (39%)	61 (34%)		
GDS				
<5	139 (77%)	135 (75%)	0.05	χ²(1)=0.27
≥5	41 (23%)	45 (25%)		
TICS				
>31	156 (87%)	156 (87%)	0.001	χ²(1)=0.00
≤31	24 (13%)	24 (13%)		
ASA class				
≤2	105 (58%)	105 (58%)	<0.001	χ²(1)=0.00
≥3	75 (42%)	75 (42%)		
Surgical site				
Hip	54 (30%)	52 (29%)	0.15	χ²(2)=2.92
Knee	55 (31%)	45 (25%)		
Spine	71 (39%)	83 (46%)		

**Table 4:** Association of variables associated with the occurrence of POD within the first three days following surgery after propensity score matching for first-generation antihistamine use POD - postoperative delirium; GDS - Geriatric Depression Scale; TICS - Telephone Interview for Cognitive Status; ASA - American Society of Anesthesiologists

Variable	No POD (n=273)	POD (n=87)	Univariable OR (95% CI; z, p)	Multivariable OR (95% CI; z, p)
Age, median (IQR)	70 (67-74)	71 (68-75)	1.03 (0.98-1.08; z=1.25, p=0.21)	-
Gender				
Female	169 (62%)	60 (69%)		
Male	104 (38%)	27 (31%)	0.73 (0.43-1.21; z=-1.20, p=0.23)	0.72 (0.42-1.21; z=-1.23, p=0.22)
GDS				
<5	219 (80%)	55 (63%)		
≥5	54 (20%)	32 (37%)	2.36 (1.39-3.99; z=3.29, p=0.001)	-
TICS				
>31	243 (89%)	69 (79%)		
≤31	30 (11%)	18 (21%)	2.11 (1.10-3.99; z=2.33, p=0.02)	-
ASA class				
≤2	169 (62%)	41 (47%)		
≥3	104 (38%)	46 (53%)	1.82 (1.12-2.97; z=2.33, p=0.02)	-
Surgical site				
Hip	87 (32%)	19 (22%)		
Knee	76 (28%)	24 (28%)	1.45 (0.74-2.87; z=1.06, p=0.29)	1.49 (0.76-2.96; z=1.15, p=0.25)
Spine	110 (40%)	44 (51%)	1.83 (1.01-3.42; z=1.96, p=0.05)	1.84 (1.01-3.45; z=1.96, p=0.05)
Perioperative antihistamine use				
No	132 (48%)	48 (55%)		
Yes	141 (52%)	39 (45%)	0.76 (0.47-1.23; z=-1.10, p=0.27)	0.79 (0.48-1.28; z=-0.97, p=0.33)

## Discussion

This is one of the rare studies to examine the perioperative in-hospital use of TCAs and first-generation antihistamines and the association with POD [7,8]. We did not observe a statistically significant association between perioperative TCA or first-generation antihistamine use and the occurrence of POD within the first three postoperative days in older surgical patients undergoing major arthroplasty or spine surgeries.

In the general population, medications with potent anticholinergic activity are used by nearly 10% of older patients (≥65), and some data suggest that their use has been increasing over time [26]. Additionally, nine major surgeries for every 100 older patients take place every year, and the perioperative physician will increasingly face the decision of whether it is safe to continue or initiate these medications during the perioperative period [27]. Given the association of anticholinergic medications with cognitive deficits, these medications have strong recommendations for avoidance in older patients during the perioperative period, with a concern for precipitating POD [8,28,29]. However, while the association of these medications in the outpatient and intensive care setting with delirium is described, there is a paucity of data regarding their use during the perioperative period and the incidence of POD [30]. Here, we present an analysis of two classes of medications with potent anticholinergic activity, TCAs and first-generation antihistamines, and the incidence of POD.

Comparison to previous studies

Prior research has mainly focused on the relationship between a composite score or index of perioperative anticholinergic drug burden, and not specific medications or classes of medications, and the incidence of postoperative delirium in older surgical patients. The literature is contradictory regarding the association of anticholinergic agents (often reported as an anticholinergic burden) and the association of POD [31-34]. Interestingly, Heinrich et al. showed that the association was only significant for patients with cognitive impairment, suggesting that certain populations may have a pre-existing sensitivity to the POD effects of these agents [35]. Recent studies examining composite anticholinergic burden scores have reported significant associations between high cumulative burden and POD, with adjusted odds ratios exceeding five in some cohorts [36]. Although patients in our study had similar preoperative cognitive status after propensity matching, our findings suggest that patients with lower TICS scores had a higher incidence of POD, providing further evidence for Heinrich et al.'s conclusion that cognitive impairment increases the risk for POD [35]. However, after adjusting for TICS score, we did not find a significant association between anticholinergic drug exposure and the incidence of POD. Duprey et al. was one of the first known studies to assess the relation between in-hospital perioperative medication use and POD in older surgical patients undergoing noncardiac surgery. However, of the medication classes assessed, only antipsychotics, benzodiazepines, and opioids were found to be associated with POD onset [34]. In a previous study, our group examined midazolam premedication prior to surgery in older surgical patients undergoing noncardiac surgery and found no association with the incidence of POD [37].

Delirium has been a well-described adverse effect of TCA use, with TCAs showing the highest rates of drug-induced delirium in a multicenter observational study of psychiatric hospital patients [38,39]. However, the relationship between perioperative TCA use and the incidence of POD remains poorly studied. TCA-induced out-of-hospital delirium has been a well-described phenomenon, but these cases usually involved an overdose or rapid dose change [40]. Whether or not patients on stable doses of TCAs admitted to the hospital following surgical procedures are at increased risk of POD has not been studied. While no prior study has examined in-hospital perioperative TCA use and POD, Heinrich et al. evaluated the preoperative use of PIMs (defined by the PRISCUS and European list of potentially inappropriate medications, EU(7)-PIM lists) in relation to the development of POD in older adults. Among patients using medications on the PRISCUS list, there was an 18.2% use of amitriptyline. There were no significant associations between PIM use and POD incidence [35]. However, all PRISCUS medications were grouped together, and TCAs were not analyzed independently. Our data found no association between the perioperative administration of TCAs and the incidence of POD within the first three days. These findings are different from the current American Geriatrics Society practice guidelines regarding the use of TCA in older patients, which recommend against any use during the perioperative period for older patients [8].

Similarly, first-generation antihistamines are also listed in the Beers criteria list to be avoided in older patients [7]. A recent systematic review by Reisinger et al. suggests that while first-generation antihistamines may be associated with an increased risk of delirium, evidence remains inconsistent and of low quality, with a meta-analysis of two case-controlled studies showing an insignificant effect measure [41-43]. Our study is the rare study to assess in-hospital perioperative first-generation antihistamine administration and POD incidence, with no significant association found. Interestingly, the point estimate suggested a protective direction (OR: 0.79), although this was not statistically significant. This may reflect confounding by indication, as antihistamines may have been administered for symptoms such as nausea or insomnia, and patients receiving them may have differed in unmeasured ways from controls.

Due to the disparate outcomes in previous research and the imprecise, non-standardized characterization of anticholinergic burden, the generalizability of published findings is limited. Consequently, additional research is necessary to elucidate the correlation between anticholinergic burden and the onset of POD. It is important to consider if the anticholinergic properties of these medications truly contribute to neurotransmitter imbalance or neuroinflammation with resultant POD, or if there are other mechanisms present that have a greater impact on POD incidence. In addition, it is also important to distinguish if there is a difference between a chronic anticholinergic burden from medications taken prior to surgery versus an acute anticholinergic challenge from starting these medications in the perioperative period. We were unable to distinguish between continuation of chronic stable dosing and new perioperative initiation. Chronic users may develop tolerance to anticholinergic effects, whereas acute dose escalation or new initiation may confer higher delirium risk. More detailed data on TCAs and first-generation antihistamines are needed to answer these questions.

Clinical implications

Few studies have specifically explored the use of Beers criteria medications perioperatively and the development of POD in older surgical patients [34,37]. Our present results suggest that while practice guidelines are helpful, an evidence-based approach is critically needed to address the use of specific classes of medications in the perioperative period for the older adult. The decision to administer any medication is always a balance between risk and benefit, and over-avoidance of medications based on poor-quality evidence may not be indicated. While our findings do not demonstrate a statistically significant short-term association with POD, they should not be interpreted as definitive evidence of safety. Decisions regarding continuation or initiation of these medications in older adults should remain individualized, weighing potential benefits against theoretical and observed risks.

Potential limitations

There are several potential limitations in our study. First, since this is a secondary analysis of data, there are general concerns such as a lack of direct control over study design and data collection from the included prospective trials, potential missed important confounders, and potential lack of generalizability. Second, the use of either TCAs or first-generation antihistamines was not randomized. While we did adjust for confounding variables using propensity score matching, there may be other factors, such as duration of surgery, depth of anesthesia, and intraoperative blood transfusions, which may have affected the incidence of delirium. In addition, the SMD between groups receiving TCAs following propensity score matching was slightly greater than 0.1 for TICS and two variables (gender and surgical site) for first-generation antihistamine use. Though these SMDs could indicate that our comparison groups were insufficiently matched, some authors suggest that any difference less than 0.2 should be considered small [44]. Therefore, we included those variables in a multivariable analysis, but found no changes to our overall conclusions. Third, the number of patients receiving TCAs was small (n=30), limiting statistical power. The observed odds ratio of 1.82 with a wide confidence interval (0.78-4.16) suggests that a clinically meaningful increase in POD risk cannot be excluded, and the analysis may be underpowered to detect modest associations. Fourth, POD incidence was only captured during the first three days, after which nearly all elective surgery patients are recovering at home, so we may have missed patients who developed POD later in their postoperative course. Similarly, we may have missed episodes of POD that developed in patients following discharge on POD 1-2, which could potentially minimize differences between control and treatment groups. Unfortunately, we were not able to include this in our analysis. Additionally, patients who were too sedated or remained intubated were excluded from delirium assessment, and these patients may have been at a higher baseline risk for POD. If so, exclusion of these individuals could bias results toward the null. Fifth, exposure was defined dichotomously (any use vs no use), and we did not capture dose, frequency, or cumulative anticholinergic burden. It is possible that higher cumulative exposure or repeated dosing may be associated with increased POD risk, and our binary exposure definition may have obscured a dose-response relationship. Additionally, because exposure and outcome were assessed within the same postoperative window, we cannot definitively establish temporal ordering for all cases. In some patients, antihistamines may have been administered on the same day that delirium was detected. Sixth, the data from our study is limited to patients who underwent elective major arthroplasty or spine surgeries. These results may not be generalizable to patients undergoing different or urgent types of surgery. Finally, secondary analysis of data, despite robust attempts to control for factors, can only yield limited conclusions. Ideally, a randomized controlled trial would be conducted to verify the conclusions suggested by our results; however, the feasibility of such a trial may be low. Finally, although we did not observe a short-term association with POD, emerging evidence suggests that perioperative anticholinergic exposure may have longer-term cognitive consequences, including incident dementia, which were not assessed in our study.

## Conclusions

In summary, our data did not demonstrate a statistically significant association between perioperative administration of TCAs or first-generation antihistamines and POD within the first three postoperative days; however, given limitations, including sample size and exposure characterization, clinically meaningful effects cannot be excluded.
